# Circular RNAs Co-Precipitate with Extracellular Vesicles: A Possible Mechanism for circRNA Clearance

**DOI:** 10.1371/journal.pone.0148407

**Published:** 2016-02-05

**Authors:** Erika Lasda, Roy Parker

**Affiliations:** Department of Chemistry and Biochemistry & Howard Hughes Medical Institute, University of Colorado, Boulder, Colorado, United States of America; Gustave Roussy, FRANCE

## Abstract

Backspliced circular RNAs (circRNAs) are prevalent in many eukaryotic systems and are spliced from thousands of different genes. Where examined, circRNAs are often highly stable and the mechanisms by which cells degrade and/or clear circRNAs from the cells are unknown. Here we investigated the possibility that cells can eliminate circRNAs into extracellular space, possibly within released vesicles such as exosomes and microvesicles. From three different cell lines and examining multiple circRNAs, we show that extracellular vesicle (EVs) preparations recovered from cell culture conditioned media contain established circRNAs. Moreover, these circRNAs are enriched over their linear counterparts within EV preparations when compared to the producing cells. This supports the idea that expulsion from cells into extracellular space, as by EVs release, can be a mechanism by which cells clear circRNAs. Moreover, since EVs can be taken up by other cells, excreted circRNAs could contribute to cell to cell communication.

## Introduction

Circular RNAs (circRNAs) can be formed by backsplicing events in which a downstream 5’ splice site is joined to an upstream 3’ splice site. Deep RNA sequencing and clever non-linear mapping strategies have revealed that higher eukaryotic cells generate thousands of different circRNAs, albeit primarily at a low level [1–5]. Despite this, little is known about how cells regulate circRNAs. It is clear that circRNA expression is dynamic, displaying varying levels and ratios to the corresponding linear RNAs in different cell types and developmental stages [6–8].

One interesting issue is how circRNAs are degraded and/or cleared from the cell. Because circRNAs lack 5' and 3' ends, they are inherently resistant to the major enzymes of mRNA degradation, which predominantly target the 5' and 3' termini [9]. Consistent with this resistance to major mRNA degrading enzymes, circRNAs are highly stable—much more so than the corresponding linear RNAs, with half-lives that can be longer than 48 hours [3,10]. One possible way that cells could clear circRNAs is to eliminate them via extracellular vesicle release.

Extracellular vesicles (EVs) are membrane-bound vessels that are released from cells and can contain cargo both internally and embedded in the membrane. These vesicles can carry a myriad of cellular components, including proteins, lipids, and RNA, despite their small size. Various populations of EVs are categorized based on size and manner of origin. For instance, exosomes originate from the fusion of endocytic multivesicular bodies with the plasma membrane, releasing the endocytosed contents to the external environment. They are generally considered to be 30–100 nm in size. Shedding microvesicles are shed directly from the plasma membrane and range in size from 100–1000 nm [11,12]. EVs can be taken up by other cells, sharing contents, and as such are a means of cell to cell communication. They may also serve as a way to dispose of unneeded cell components [12]. Cell-free RNA could also be complexed with proteins in extracellular RNPs independent from vesicles [13].

In this study, we addressed the possibility that cells clear cytoplasmic circRNAs through the release of cargo-bearing EVs, including exosomes and microvesicles. We show that cultured cells secrete EVs, and that RNA isolated from EV preparations contain circRNAs. Furthermore, circRNAs are enriched in EV preparations over their linear counterparts (conventionally spliced RNAs originating from the same gene) compared to the producing cells. Thus, extracellular expulsion, as in EVs, is one way that cells can eliminate circRNA build-up. Intriguingly, it may also be a way for cells to transfer functional circRNAs as a means of communication.

## Results

**Fig 1:**
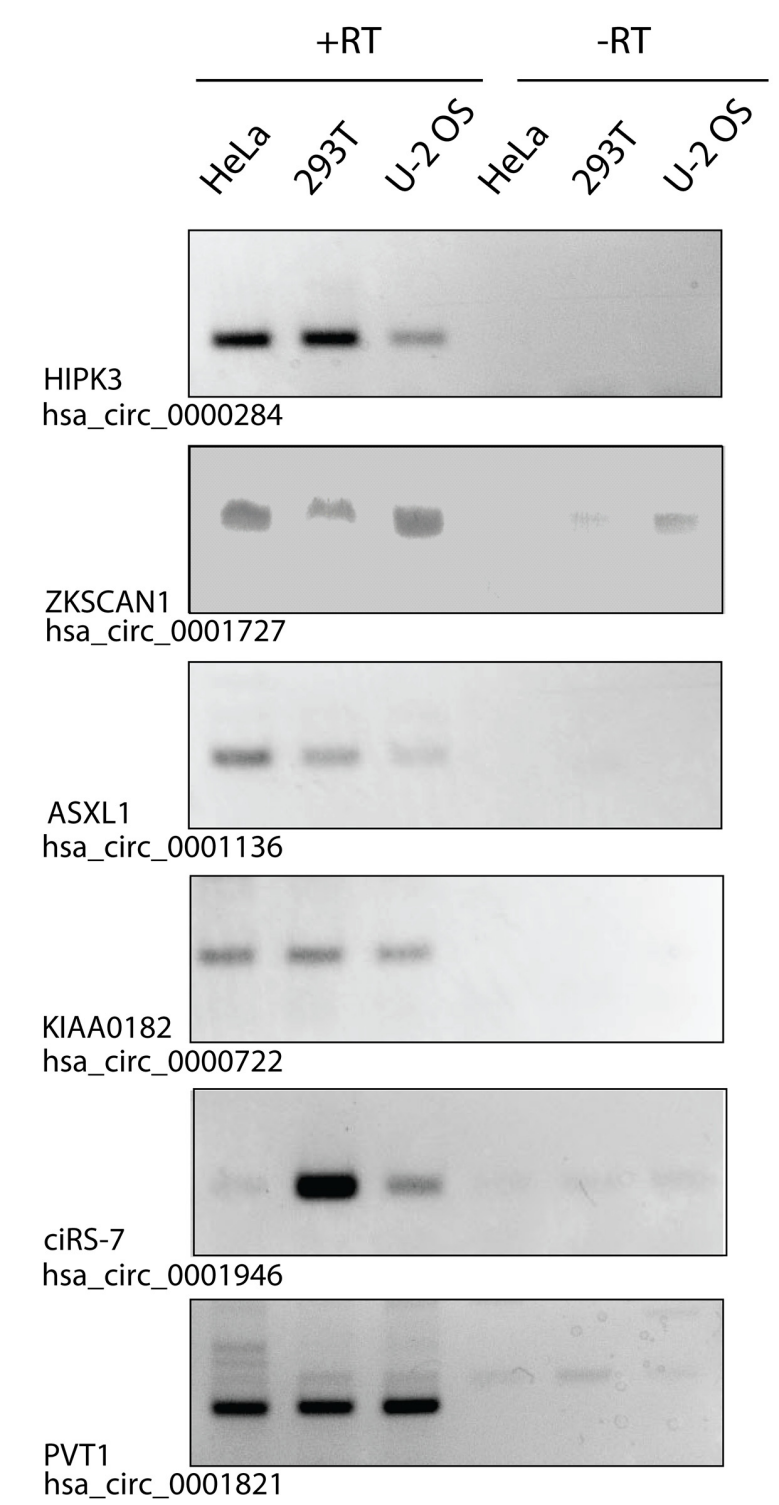
circRNAs are detected in preparations enriched in extracellular vesicles. Preparations enriched in EVs were precipitated from conditioned media from HeLa, 293T, or U-2 OS cultured cells. Isolated RNA was used for RT-PCR with divergent primers to detect indicated backspliced circRNAs. Products were visualized by ethidium bromide on an agarose gel. A selection of previously identified circRNAs were chosen for analysis. Gene names and circRNA IDs shown are according to the circBase database (http://circbase.org/) [15]. -RT lanes omitted reverse transcriptase.

To determine if EV preparations contain circRNAs, we isolated EVs from the conditioned culture media of HeLa, 293T, and U-2 OS cells. In order to analyze EVs in a size range that would include both exosomes and microvesicles, we used the ExoQuick method to purify EVs (SBI)[14], which is reported to precipitate vesicles of 60–180 nm. A selection of circRNAs were chosen for analysis based on reported abundance and detection in cultured cell lines using datasets and annotations compiled in circbase (http://circbase.org/) [15], some of which had been validated in other studies [3,16] (see Materials and Methods for circbase IDs and primer sets). Outward-facing (divergent) primers were then used to detect five previously identified circRNAs by RT-PCR. All PCR products were of expected sizes on agarose gels (Fig 1) according to previously described backsplicing exon boundaries [15], and were cloned and sequenced to verify backspliced junctions.

Strikingly, we detected all six circRNAs in EV preparations (Fig 1). Moreover, the circRNAs were detected in EVs from all three cell lines. This indicates that EV circRNA is a common property of many cell types, although we did observe some cell-line specific differences in the amount of each circRNA detected in the EVs. This implies that circRNAs are released from different cell types.

**Fig 2:**
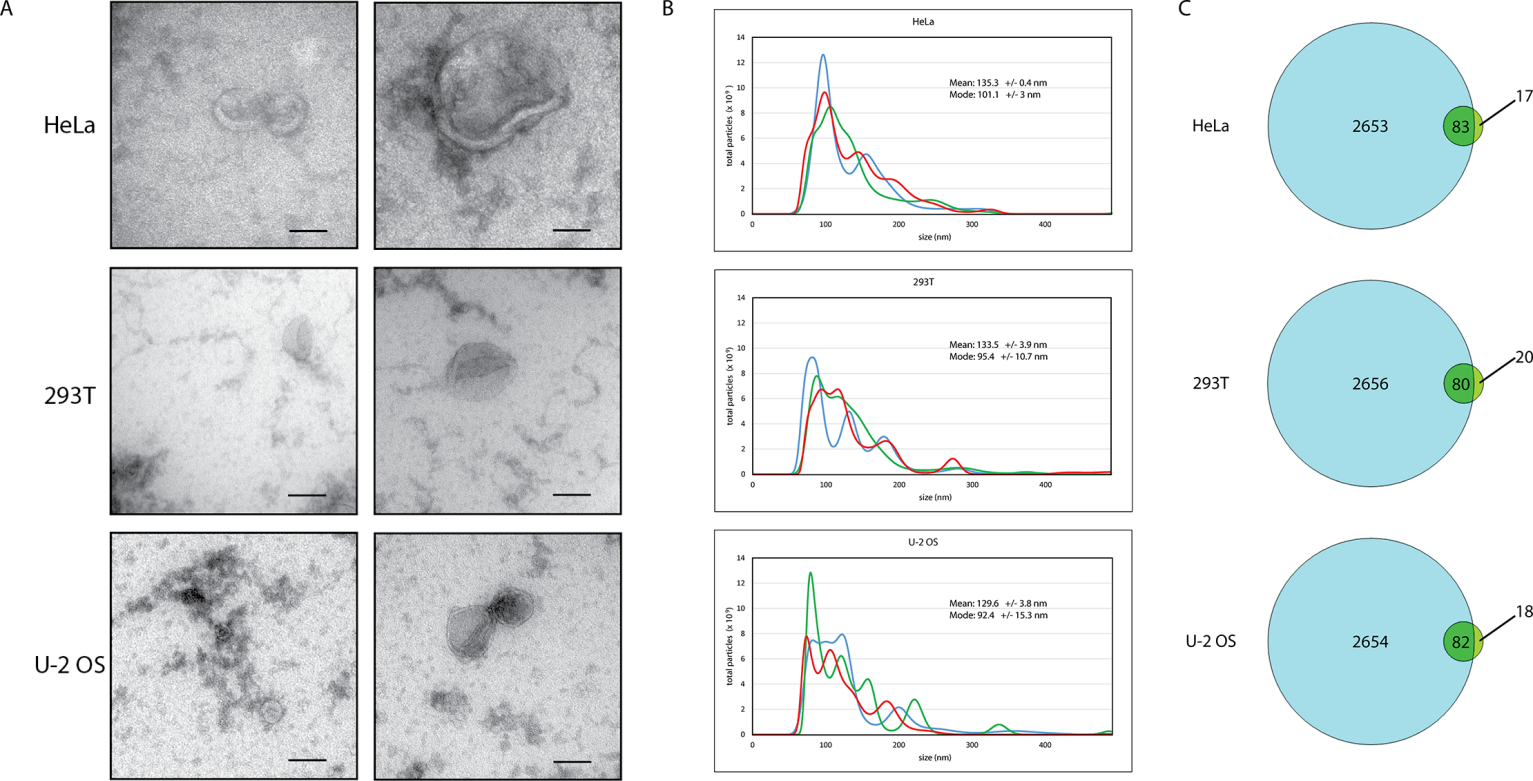
Precipitated preparations from conditioned cell culture media from HeLa, 293T, or U-2 OS cells show characteristic particle morphology, size, and protein composition of extracellular vesicles, including exosomes (30–100 nm) and microvesicles (100–1000 nm). (A) Electron microscopy of vesicles with a typical cup-shaped morphology in a variety of sizes consistent with exosomes and microvesicles. Representative examples of smaller and larger vesicles are shown. Scale bars are 100 nm. (B) Nanoparticle Tracking Analysis profiles of particle size and distribution in each sample are characteristic of small extracellular vesicle preparations containing exosomes and microvesicles. Different colored traces represent three different video captures of the same sample. (C) Venn diagram of the list of GO term “extracellular exosome” (GO:0070062) cellular component proteins (2736 proteins, blue circles) compared to the list of the top 100 most abundant proteins (green circles) identified by mass spectrometry analysis of each EV precipitated sample. The number of overlapping proteins is indicated within each intersecting region.

In order to conclude that the circRNAs we detect were really associated with EVs, we used three measures to validate that our samples displayed characteristics of EVs. Such verification is particularly important since extracellular media may contain a variety of non-vesicular macromolecules, including lysed cells, as well as other types of membrane-bound components (i.e. apoptotic bodies and ER fragments) [17]. Electron microscopy of each EV preparation (Fig 2A) confirms the presence of vesicles of expected sizes with the characteristic cup-shape. Although the cup-shaped appearance may be a change conferred by the methyl cellulose uranyl acetate protocol used for the microscopy, it is nevertheless a distinguishing view of exosomes treated in this way [18].

Nanoparticle Tracking Analysis (NTA), which measures particles from 10 to 2000 nm, was used to analyze the particle size distribution of our EV preparations to see if they are representative of bona fide exosome and microvesicle EVs [19,20]. We captured images using a NanoSight (Malvern) and analyzed particle size and concentration for every EV preparation. As seen in Fig 2B, representative EV preparations from three cell types contain particles of the expected sizes and distribution of exosomes (30–100 nm) and microvesicles (100–1000 nm) [11]. This indicates that the circRNAs detected in Fig 1 co-precipitate with particles of predominantly 75–200 nm from each cell type, as would be expected of ExoQuick precipitated extracellular vesicles.

As an additional verification we performed mass spectrometry on each of the EV preparations, which can be used to validate the general protein composition of an EV preparation [17,21]. This enabled identification of the protein content to complement the visual confirmation and size distribution analysis. After filtering the list to remove common cell culture contaminant proteins, 244, 227, and 232 proteins were identified in the EV preparations of Hela, 293T and U-2 OS cells respectively (S1 Table). These were analyzed for Gene Ontology term enrichment by String GO analysis for Cellular Components. This clearly shows characteristics of extracellular vesicles. The top 5 terms (sorted by p-value) for each preparation are shown in Table 1 and are all indicative of EV-enriched preparations. Similar GO term lists resulted from analysis using only the top 100 most abundant proteins from each sample. In addition, the top 100 proteins were compared against all GO term “extracellular exosome” (GO:0070062) cellular component proteins according to EMBL-EBI QuickGO Gene Ontology Terms and Annotations (http://www.ebi.ac.uk/QuickGO/GTerm?id=GO:0070062). 80%-83% of the abundant proteins identified by mass spectrometry are listed as extracellular exosome proteins (Fig 2C).

**Table 1:** Mass spectrometry GO terms Cellular Components.

HeLa (all 244)			
GO_id	Term	Number Of Genes	p-value
**GO:0070062**	extracellular exosome	180	7.43E-108
**GO:0031988**	membrane-bounded vesicle	168	4.06E-81
**GO:0031982**	vesicle	169	1.77E-80
**GO:0044421**	extracellular region part	170	4.43E-80
**GO:0005576**	extracellular region	174	2.00E-71
**293T (all 227)**			
**GO_id**	**Term**	**Number Of Genes**	**p-value**
**GO:0070062**	extracellular exosome	171	1.40E-103
**GO:0031982**	vesicle	164	2.11E-81
**GO:0031988**	membrane-bounded vesicle	161	1.25E-79
**GO:0044421**	extracellular region part	153	9.31E-68
**GO:0005576**	extracellular region	154	2.15E-57
**U-2 OS (all 232)**			
**GO_id**	Term	Number Of Genes	p-value
**GO:0070062**	extracellular exosome	179	3.20E-112
**GO:0044421**	extracellular region part	171	3.78E-86
**GO:0031982**	vesicle	166	8.92E-82
**GO:0031988**	membrane-bounded vesicle	164	3.39E-81
**GO:0005576**	extracellular region	176	1.11E-78

Top 5 gene ontology terms of cellular components of the total proteins identified in HeLa, 293T, and U-2 OS EV preparations from cultured cells indicate enrichment in small extracellular vesicle terms. Analysis using the top most abundant 100 proteins resulted in similar GO terms (not shown).

We interpret the combined electron microscopy, Nanoparticle analysis, and mass spectrometry data to indicate that our preparations are indeed enriched in extracellular vesicles of a variety of sizes, consistent with known sizes and protein compositions of exosomes and microvesicles [17]. For example, exosome tetraspanin protein CD81 and syntenin-1, a membrane-associated, EV-enriched protein are detected by mass spectrometry in the top 100 proteins of both the 293T and U-2 OS preparations. Annexins, expected to be enriched in exosomes, were identified in the HeLa and U-2 OS preparations, whereas calnexin, an ER protein, is absent in all three EV preparations. While this validates that our preparations are indeed enriched in EVs, it does not exclude the possibility that other, non-vesicle components have co-precipitated with the EVs. It is formally possible that the circRNAs detected in this study are not carried inside the membrane-bound vesicles in the EV preparations, but rather co-precipitate along with the EVs, perhaps associated with some other similar-sized RNP complex.

**Fig 3:**
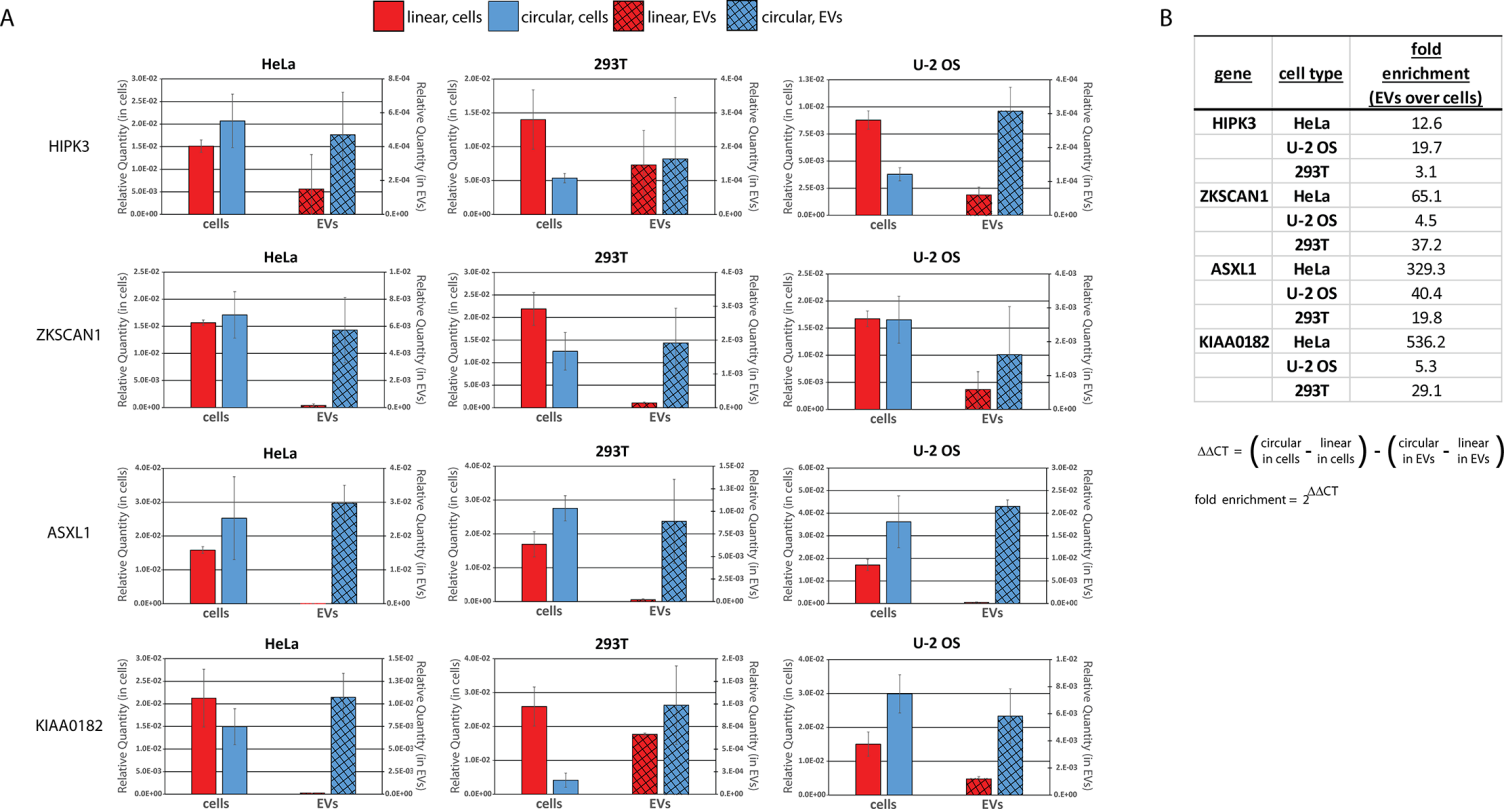
circRNAs are enriched in EV preparations over linear counterparts. RNA isolated from EV preparations and corresponding source cells was analyzed by real-time quanitative RT-PCR for indicated backspliced circRNAs and the linear spliced mRNA counterparts of the same gene. (A) The relative quantity of each RNA is shown with the level in cells plotted on the primary (left) axis and in EVs on the secondary (right) axis of each bar graph. Error bars are standard deviation of triplicate reactions. (B) Fold enrichment of circRNAs compared to the linear spliced counterpart RNAs in EVs over their corresponding source cells. Fold enrichment is calculated from the qPCR CT (threshold cycle) values for each sample according to the formula shown.

In principle, circRNAs and linear forms of the same mRNAs could be equally excreted by the cell in EVs, in which case we would expect that the ratios of these molecules in EVs should be similar to the ratios within the cell. Alternatively, if circRNA excretion in EVs is important for the clearance of these molecules from the cell, circRNAs might be preferentially packaged in EVs for excretion, which predicts there would be a greater proportion of circRNAs targeted to vesicles compared to corresponding linear RNAs. In order to test this, we compared real-time quantitative RT-PCR signals of four known circRNAs and their linear counterparts from both the EVs and their source cells (Fig 3). For each circRNA gene, the corresponding linear mRNA was analyzed by choosing a primer pair that would span an intron in a region that does not include the backspliced circRNA exon(s). EV preparations do not yield a quantifiably large amount of RNA. Nonetheless, some circ and linear RNAs could be reliably detected by RT-qPCR. circRNA ciRS-7 (hsa_circ_0001946) was not included in the qPCR analysis, as this antisense transcript to the CDR1 gene does not have a linear spliced form for comparison. circRNA PVT1 (hsa_circ_0001821) was not included due to the abundance of both circular and linear alternative spliced isoforms.

Strikingly, we observed that in all three cell types, for all circRNAs analyzed, EV circRNA was greater than the corresponding EV linear PCR, when compared to the same ratio in cells (Fig 3). As each primer set amplifies a distinct PCR product, we cannot compare precise amounts of linear versus circular RNA for each gene by this method. However, when we analyze the ratio of circular to linear RNAs within the same sample, and then compare that ratio from cellular RNA to the same ratio from EV preparation RNA, we see a clear enrichment of circRNA in EVs. For example, in HeLa cells, both circular and linear forms of ZKSCAN1, ASXL1, and KIAA0182 were readily detectable. However, EV preparations from HeLa cell media contained almost no detectable linear forms, yet fairly high levels of each circRNA (hsa_ circ_0001727, hsa_ circ_0001136, hsa_ circ_0000722). Further, circHIPK3 was detected in U-2 OS cells with a lower relative quantity than linear HIPK3, yet U-2 OS EVs contained a higher relative quantity of circHIPK3. Calculating the fold enrichment of circular over linear isoforms in EVs compared to cells using the delta delta CT relative quantification calculation shown in Fig 3B, it is evident that in each case, EV circRNAs are enriched over their linear counterparts. This shows that circRNAs are enriched in EV preparations. Similar results in three different cell lines indicated that the enrichment of circRNAs in EVs over their corresponding linear form is not a cell type specific event.

## Discussion

**Fig 4:**
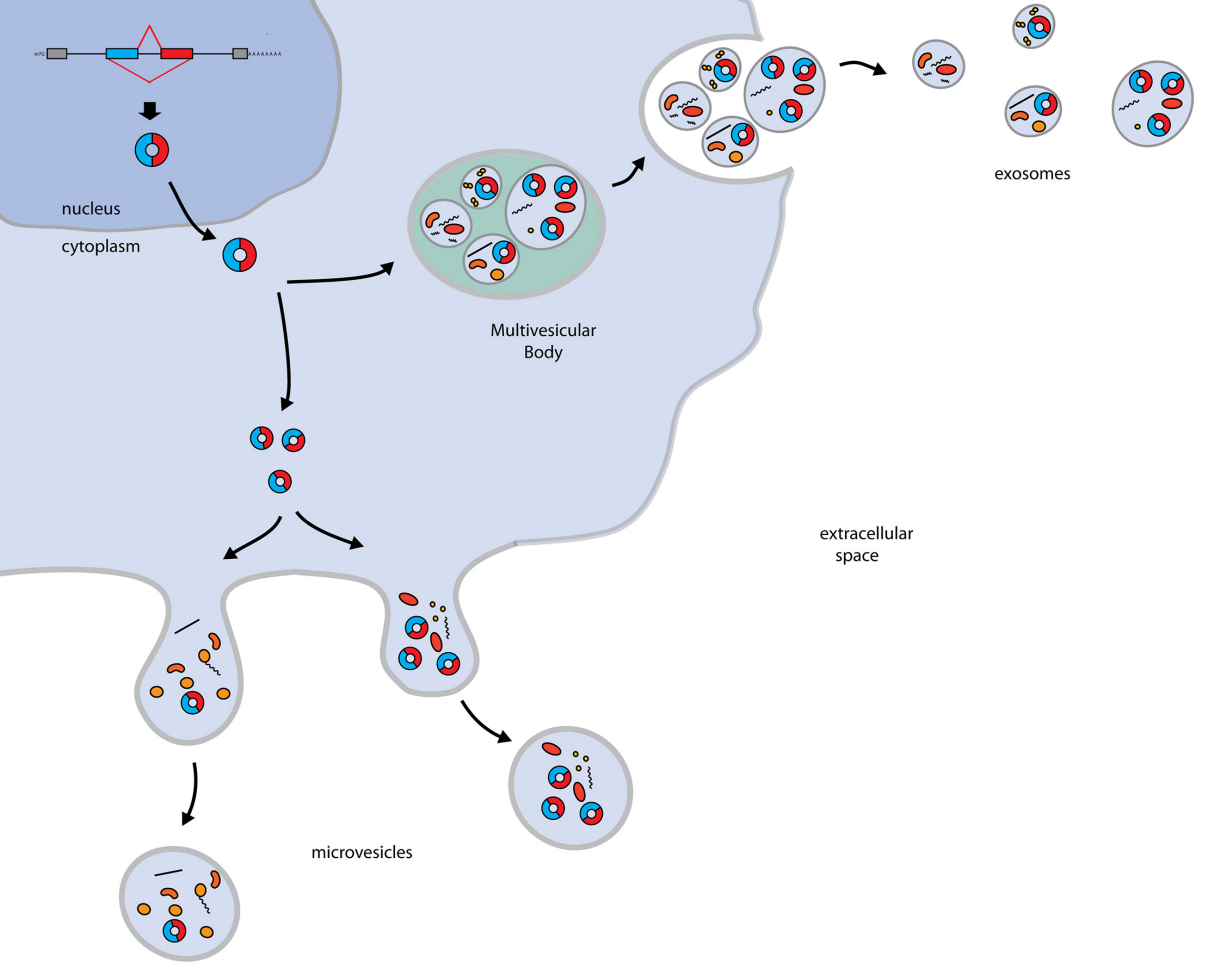
Possible mechanisms for circRNA clearance. circRNAs formed by nuclear backsplicing events are eliminated from cells by incorporation into vesicles that are released, such as exosomes or microvesicles.

While much attention has been focused on circRNA expression and biogenesis, very little is known about the metabolism of these molecules within the cell. Given their high degree of stability, and their presumed resistance to exonucleolytic degradation, circRNAs may accumulate. It is not known whether such a build-up would be toxic, but it is likely that cellular mechanisms exist to control circRNA levels. Here, we show that one way that cells could deal with circRNAs is to excrete them via extracellular vesicles (Fig 4). The key evidence for this conclusion is that circRNAs are easily detected in EV preparations and circRNAs are enriched in EVs as compared to their levels in the cell body relative to linear forms of the same genes. Interestingly, while this work was in preparation, a recent paper has also provided evidence for circRNA enrichment in EV preparations indicating the generality of this result [22]. Thus, we conclude that one aspect of reducing the accumulation of circular RNAs due to backsplicing is their preferential export from the cell.

It is likely that additional mechanisms exist to degrade or expel circRNAs, either involving other vesicle trafficking mechanisms, or endonuclease cleavage. For example, the circRNA ciRS-7 /CDR1as is subject to miRNA-mediated cleavage when associated with one particular miRNA. However, this is based on particular sequences [23], and would not likely apply to the thousands of other circRNAs.

Intriguingly, *ciRS-7 /CDR1as* is also detected in EV preparations. An additional possibility is that functional circRNAs in some cases might be packaged into EVs for the purpose of cell to cell communication. *ciRS-7* /*CDR1as* can function as a microRNA sponge, competing with endogenous miRNA targets and “sponging” up particular miRNAs [4,24]. Perhaps in EVs it functions to relay the miRNA status of the source cells to other cells. Although this function is based on specific sequences of this gene and is likely not a common function for all circRNAs, it is nevertheless interesting to speculate that stable circRNAs carry messages to other cells via vesicles.

## Materials and Methods

### Cell Culture

HeLa, 293T, and U-2 OS (ATCC HTB-96) cells were maintained at 37°C, 5% CO_2_, in DMEM media supplemented with 10% fetal bovine serum, 2 mM L-Glutamine, 1% Pen/Strep (100 U/mL penicillin, 100 μg/mL streptomycin), and 1mM sodium pyruvate. For EV collection, cells were seeded at 1–2 x 10^4^ cells/cm^2^ in DMEM media supplemented with 10% exosome-depleted FBS (Exo-FBS, SBI), L-GlutaMAX, 1% penicillin/streptomycin, and sodium pyruvate. After 3 days, conditioned culture media was collected for EV isolation and corresponding cells were collected and resuspended in PBS for RNA isolation.

### EV Isolation

Conditioned culture media was centrifuged for 30 minutes at 3000 x g, 4°C in conical tubes. 0.2 x volume ExoQuik-TC (SBI) or equivalent reagent was added to the supernatant, mixed, and incubated at 4°C overnight. Samples were then centrifuged 30 minutes at 1500 x g, 4°C, media was aspirated to 500 μL, and centrifuged again for 30 minutes at 1500 x g, 4°C. Remaining media was aspirated, and EV pellets were resuspended in PBS.

### Electron Microscopy of EV Samples

EV samples were prepared and visualized by electron microscopy by the University of Colorado Boulder Electron Microscopy Service. Samples resuspended in PBS were treated essentially as described in [18] with modifications. Briefly, 5–10 μl of the PBS/exosome containing solution was deposited on parafilm. A glow-discharged, carbon coated EM grid was placed carbon side down on the drop of solution and membranes were allowed to adsorb for 20 minutes. Grids were washed once on 50–100 μL drops of PBS (on paraflim) for 1–2 minutes each, then 4–8 times on 50–100 μL drops of water for 1–2 minutes each. Grids were contrasted on a 20 μL drop of 2% uranyl-acetate for 1–2 minutes, then transferred to a 20 μL drop of 4% uranyl-acetate, 2% methyl-cellulose, and let stand for 10 minutes on ice (glass dish covered with parafilm on ice). Excess methyl-cellulose-UA was removed by dragging grids over filter paper with the loop tool and allowed to dry completely before visualization on the microscope.

### RT-PCR

EV and total cell RNA was isolated by Trizol LS (Life Technologies) according to the manufacturer’s recommendations. RNA was used in a 40μL RT reaction with SuperScript III Reverse Transcriptase (Life Technologies) and 150ng random 9-mer primers. For Fig 1, 2μL EV cDNA was used in a 40μL PCR reaction of 38 cycles with GoTaq (Promega), and outward facing (divergent) primers for backspliced circRNA detection. 35μL of each reaction was run on a 2% agarose gel and stained with ethidium bromide. All circRNA RT-PCR products were cloned and sequenced and verified expected, previously described exon boundaries and backspliced junctions. The circRNAs chosen for analysis are listed in Table 2.

**Table 2:** circRNAs used in this study.

Gene symbol	circRNA ID	circRNA position	alternate gene symbol
HIPK3	hsa_circ_0000284	chr11:33307958–33309057	
PVT1	hsa_circ_0001821	chr8:128902834–128903244	TCONS_00015354
ASXL1	hsa_circ_0001136	chr20:30954186–30956926	
KIAA0182	hsa_circ_0000722	chr16:85667519–85667738	GSE1
ciRS-7	hsa_circ_0001946	chrX:139865339–139866824	CDR1
ZKSCAN1	hsa_circ_0001727	chr7:99621041–99621930	

circRNAs chosen for analysis in this study are listed according to annotation in circbase (http://circbase.org/) [15] by gene symbol and circRNA ID number.

For Fig 3, cDNA was diluted 1:2 for EVs and 1:100 for cells (although replicates using other dilutions gave similar results), and 1μL diluted cDNA was used in triplicate 10μL qPCR reaction using iQ SYBR Green Supermix (Bio-Rad #170–8882) and primer pairs listed below for detection of backspliced circRNA or linear spliced RNA from each gene. Linear primer pairs span an intron in a region that does not include the backspliced circRNA exon(s). The primer sets used for each target gene are listed in Table 3. Relative starting quantity was calculated for each reaction according to a standard curve for each primer set.

**Table 3:** Target genes and primers used in the study.

Gene symbol	ID	primer 1	primer 2
HIPK3	hsa_circ_0000284	GTCGGCCAGTCATGTATCAA	TGGAATACACAACTGCTTGGC
ZKSCAN1	hsa_circ_0001727	CCTCGAGCTTTGACCTTCATCACG	CTCACCTTTATGTCCTGGGAGGT
ASXL1	hsa_circ_0001136	GGACTTCCCCTCTCGCATG	TCCTTCTGCCTCTATGACCTG
KIAA0182	hsa_circ_0000722	GCGCCATCCTCCAGCTTTG	GGTCGCGGTGGAAAGCATC
CiRS-7 / CDR1-as	hsa_circ_0001946	ACCCAGTCTTCCATCAACTG	GACACAGGTGCCATCGGA
PVT1	hsa_circ_0001821	AAGACCCCGACTCTTCCTGG	TTCCACCAGCGTTATTCCCC
HIPK3	linear HIPK3	GCCACTACAGATCCGACCAG	TGCGGCATCACTGAGTTATAATG
ZKSCAN1	linear ZKSCAN1	ATGAGGGTAGTCCCAGAGACC	CTGAGATTCCTCCGAGCCAG
ASXL1	linear ASXL1	TCCCAGGGACAGCTACAGAG	CCCGTTTACCTTCAGAGGAGT
KIAA0182	linear KIAA0182	CCGCTACAGCCCTGATGAGA	CTTCATGGCACGGAGCATTTC

Primer pair sequences for PCR analysis of each of the listed target genes.

### Nanoparticle Tracking Analysis

EV samples were diluted 1:1000 in PBS. Particles were analyzed using the NanoSight NS300 (Malvern) with syringe pump, a 532 nm laser, and a sCMOS camera. Three videos of 30 seconds were collected for each sample and analyzed by NTA 3.0 software. Control samples of 100 nm and 200 nm polystyrene microbeads were analyzed in parallel (not shown).

### Mass Spectrometry of EV Samples

EV samples were lysed in 4% SDS, 0.1 M Tris, and proteins were analyzed by LC-MS/MS at the Central Analytical Mass Spectrometry Facility at the University of Colorado Boulder. Data were searched against the proteome database using the MaxQuant/Andromeda platform, and then filtered to remove common cell culture contaminant proteins using the MaxQuant database for contaminants, leaving a total list of 244, 227, and 232 identified proteins from HeLa, 293T, and U-2 OS samples, respectively. These proteins were ranked according to relative abundance calculated by spectral counts/molecular weight (S1 Table). These lists were analyzed with the online tools of String (http://string-db.org/), using the entire list of proteins or the 100 most abundant proteins for each sample. Enrichment GO analysis was performed using gene ontology terms of cellular components. These lists were also compared to the list of GO term “extracellular exosome” (GO:0070062) cellular component proteins according to EMBL-EBI QuickGO Gene Ontology Terms and Annotations (http://www.ebi.ac.uk/QuickGO/GTerm?id=GO:0070062).

## Supporting Information

S1 Table Proteins identified by mass spectrometry of EV samples from HeLa, 293T, and U-2 OS samples, ranked according to relative abundance. (XLSX)
